# Endogenous Multilayer Control of Cambial Stem Cells and Its Consequences for Wood Formation

**DOI:** 10.3390/plants15050710

**Published:** 2026-02-26

**Authors:** Yun-Jing Bao, Fang-Jing Fan, Ying-Gao Liu, Fu-Yuan Zhu

**Affiliations:** 1 State Key Laboratory for the Development and Utilization of Forest Food Resources, State Key Laboratory of Tree Genetics and Breeding, The Southern Mordern Forestry Collaborative Innovation Center, College of Life Sciences, Nanjing Forestry University, Nanjing 210037, Chinafanfangjing@njfu.edu.cn (F.-J.F.);; 2 College of Life Science, Shandong Agricultural University, Taian 271018, China

**Keywords:** vascular cambium, wood formation, phytohormone, secondary xylem

## Abstract

The vascular cambium serves as the fundamental meristem for wood formation. It determines wood biomass and structural properties by balancing self-renewal with the bidirectional production of xylem and phloem. This process is controlled by a complex network of peptides, transcription factors, and phytohormones. These regulatory networks coordinate cambial stem cell activity, balancing cell division and differentiation. Additionally, layers of regulation such as chromatin state, protein stability, and non-coding RNAs add significant complexity to these networks. Emerging single-cell and spatial transcriptomics, together with quantitative modeling, now resolve cambial heterogeneity, predicting the dynamic characteristics of wood formation. This review synthesizes current knowledge of cambial regulation, highlighting how feedback loops, spatial gradients, and dynamic signaling networks collectively orchestrate the predictive potential for improving cambial activity and wood formation.

## 1. Introduction

Wood formation is sustained by secondary growth driven by the vascular cambium, which is essential for plant fitness by providing mechanical support and facilitating long-distance transport. Additionally, wood supports diverse industrial applications, including construction, paper production, and bioenergy [1]. Wood development proceeds through coordinated modules, including the maintenance of cambial cells, differentiation of xylem/phloem precursor cells, cell expansion, secondary cell wall (SCW) thickening, and programmed cell death [2,3]. The rate and spatial precision of cambial regulation determine the upstream supply of differentiating cells, driving radial expansion to produce thicker stems and roots, and establishing efficient vascular transport systems that support sustained growth [4,5,6,7]. This positions the regulation of cambial activity at the foundation of secondary growth.

Plant meristems are classified into apical meristems, which drive longitudinal growth, and lateral meristems, which drive radial growth [8]. The vascular cambium serves as the key tissue for secondary vascular development in woody plants [9]. It is a cylindrical lateral meristem formed by the fascicular cambium and the interfascicular parenchyma cells that close into a spatially continuous cylinder along the stem axis [9,10]. Through coordinated periclinal and anticlinal divisions, cambial cells balance self-renewal with bidirectional output. Specifically, periclinal divisions drive radial growth while anticlinal divisions ensure circumferential continuity as the stem increases in diameter [11]. This precise orientation of cell divisions allows the cambium to maintain its structural integrity while supplying the precursors for wood development [12]. Therefore, endogenous signals are particularly important for localizing and regulating cambial activity in woody species.

**Figure 1:**
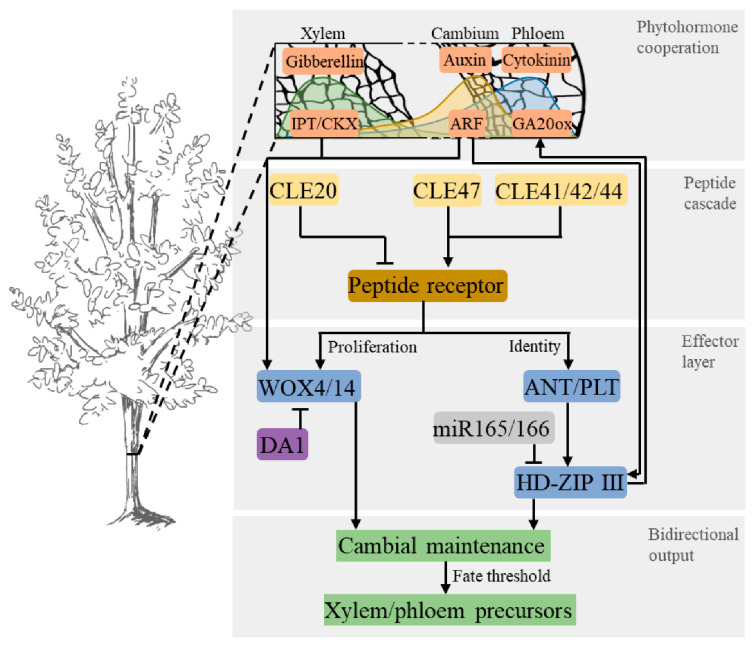
Multilayer endogenous control of the vascular cambium. Top panel shows phytohormone gradients across the radial axis: auxin peaks in the cambium, cytokinin is biased toward phloem, and gibberellin toward xylem. Middle panel shows peptide cascade and effector layer: phloem-derived *CLE41*/*42*/*44*, cambium-derived *CLE47*, and xylem-derived *CLE20* feed into a peptide receptor hub, which branches into downstream modules. On the one hand, proliferation arm *WOX4*/*14* promotes cambial divisions but is restrained by the DA1-family ubiquitin pathway. On the other hand, identity arm *ANT*/*PLT* maintains stem cell state and positions fate thresholds, as *miR165*/*166* limits *HD*-*ZIP III* dosage to set domain width. In parallel, phytohormone cooperation integrates auxin, cytokinin, and gibberellin inputs to cooperate with transcriptional factors and execution of periclinal divisions. Bottom panel shows that these modules together sustain cambial maintenance and balance bidirectional outputs of xylem and phloem precursors. Arrows indicate activation; blunt lines indicate inhibition; and colors denote layers: phytohormones (orange), peptides (yellow), receptor (amber), transcription factor (blue), post-translational control (purple), miRNA (grey), and outputs (green).

Recent research highlights three interacting regulatory layers related to cambial development, including peptide receptor signaling [13], transcriptional control [14], and phytohormone integration [15]. These layers work together to regulate cambial activity, ensuring a balance between stem cell proliferation, differentiation, and maintenance, which ultimately determines the output of xylem and phloem. Unlike primary meristems, which are primarily governed by intrinsic developmental programs, cambial stem cells are uniquely regulated by the integration of dynamic feedback loops, precise temporal control, and environmental cues [16,17]. This complex regulatory network facilitates secondary growth and enables the continuous and adaptive wood formation throughout the lifespan of a tree. We propose a conceptual model that illustrates how these regulatory pathways jointly define the cambial stem cell zone and determine bidirectional wood output (Figure 1). While these pathways are widely conserved, their interaction within the cambium is uniquely tailored to meet the specific requirements of secondary growth. Integrating these layers provides insights into how cambial stem cells maintain their identity, which informs predictive breeding and the rational engineering of biomass and material properties.

In recent years, model species such as *Arabidopsis* have provided significant contributions to understanding the molecular mechanisms underlying cambial activity, particularly during primary growth. These models have been instrumental in defining the signaling networks and phytohormonal pathways that control stem cell fate, division, and differentiation within meristems. Current research has highlighted conserved molecular frameworks shared between herbaceous and woody plants, such as the core *CLE*-*WOX* signaling modules [18,19]. However, there remain limitations in applying these findings directly to woody plants due to the complexities of secondary growth, the prolonged lifespan of tree species, and various environmental influences [20,21,22]. While many conserved mechanisms have been identified, particularly in juvenile plants like *Populus*, these findings are not fully applicable to mature woody plants as structural characteristics differ [23]. Specifically, mature woody plants possess more expansive secondary vascular systems and higher degrees of lignification maintained over decades [24]. Furthermore, while core components like *CLE* peptides and *WOX* transcription factors are evolutionarily conserved, their regulatory mechanisms and functional integration often diverge [25]. These functional adaptations highlight the necessity of integrating findings from both model species and mature woody plants to develop a more comprehensive understanding of cambial activity and wood formation.

In this review, we synthesize evidence on cambial regulation with an emphasis on woody plants, using *Arabidopsis* as a reference. We discuss peptide receptor signaling, transcriptional modules, and phytohormone integration. Furthermore, we explore how emerging single-cell and spatial transcriptomic approaches resolve cambial regulation at cellular resolution, which might guide future research into the vascular cambium.

## 2. Maintenance of Cambial Stem Cell Activity

### 2.1. Cross-Layer Peptide Signaling

A conserved peptide receptor system positions and sustains cambial activity through non-cell-autonomous communication from the phloem to the procambium [26]. Phloem-expressed *CLAVATA3*/*EMBRYO SURROUNDING REGION 41*/*42*/*44* (*CLE41*/*42*/*44*) genes encode proteins that are processed into *TRACHEARY ELEMENT DIFFERENTIATION INHIBITORY FACTOR* (*TDIF*) peptides. These peptides are secreted into the apoplastic space and transported across tissue layers to the procambium through apoplastic diffusion [13,27]. This apoplastic transport is facilitated by the specific cellular anatomy of the cambial zone. During active growth phases, the absence or limited presence of plasmodesmata in the cambial initial zone prevents symplastic movement. Consequently, TDIF peptides must diffuse through the extracellular space to reach and bind with receptors on their target cells [18,28]. Loss of *CLE41* reduces the number of cambial cell layers while leaving sensitivity to exogenous TDIF intact, indicating that *CLE41* constitutes the principal endogenous TDIF source, with partial redundancy from *CLE44* [13]. Notably, additional tissue-specific inputs diversify this axis. Xylem-derived *PtrCLE20* acts on the cambium through long-distance transport, inhibiting cell division and forming a negative feedback regulation [29]. In contrast, *PttCLE47* is expressed within the cambium and promotes cambial proliferation. Consistently, RNAi-mediated silencing of *PttCLE47* results in fewer cambial cell layers and a significant narrowing of the secondary xylem region, with little effect on the phloem [25]. Furthermore, overexpression of *PttCLE41* significantly increases the biomass of wood [29,30], underscoring the dosage-dependent control of radial growth. Together, the diverse tissue origins and action mode of *CLE* ligands enrich the regulatory network and provide distinct strategies for defining the cambial region.

TDIF peptides are recognized by the leucine-rich repeat receptor-like kinase *PHLOEM INTERCALATED WITH XYLEM* (*PXY*) on the plasma membrane of cambial cells, triggering a cascade of intracellular events that maintain the cambial stem cell identity [13]. Mutants of *pxy* fail to respond to TDIF and develop discontinuities in the cambial cylinder during secondary growth [31], demonstrating the essential role of this receptor in maintaining circumferential continuity.

Downstream of *PXY*, *WUSCHEL HOMEOBOX RELATED 4* (*WOX4*) is a key regulator of cambial division and acts partly redundantly with *WOX14*. Notably, *wox4*/*wox14* mutants retain residual cambial activity [14]. The rapid upregulation of *WOX4* by TDIF in a *PXY*-dependent manner highlights the integration of peptide signaling with transcriptional regulation in maintaining cambial activity. Furthermore, *wox4* mutants show reduced procambial proliferation but remain sensitive to the TDIF-mediated inhibition of xylem differentiation. Since overexpression of *WOX4* is insufficient to replicate the proliferation induced by TDIF in the hypocotyl, additional *WOX4*-independent pathways are likely required [13]. This points to a more complex network in which genetic interactions reveal parallel determinants of cambial activity. For example, cambial activity is nearly abolished in the double mutants of *wox4* and *KNOTTED*-*like from Arabidopsis thaliana 1* (*knat1*), revealing *KNAT1* alongside *WOX4* as a major determinant of division [32]. In parallel, an identity branch composed of *CAMBIUM*-*EXPRESSED AINTEGUMENTA*-*LIKE* (*CAIL*) transcription factors operates downstream of *TDIF*-*PXY*. Consistent with this, mutants of *PLETHORA 3*/*5*/*7* (*PLT3*/*5*/*7*) exhibit reduced cambial cell numbers and compromised secondary growth [33].

The *CLE41*-*PXY*-*WOX* pathway exhibits dynamic activity patterns, orchestrating cambial activity by integrating internal developmental stages with external environmental cues [34]. In woody species, the sequential phases of cambial cell development, from initial activation to peak proliferation and eventual maturation, are fundamentally synchronized with the seasonal cycle. For instance, in *Betula pendula*, the expression of *BpCLE41*/*44a* peaks during the initial proliferation phase, which coincides with the early spring reactivation of cambial initials. As development progresses, *BpPXY* expression reaches its maximum during the mid-differentiation phase, supporting the intensive mid-season production of secondary xylem. Additionally, while *BpWOX4* typically maintains a high correlation with the rate of cell division, its expression can decouple in cases of abnormal xylogenesis, leading to premature cessation of activity during late-season maturation phases [35]. This intrinsic developmental clock is further modulated by broader seasonal variations across the annual growth cycle. Similarly, in *Populus*, *WOX4-like* genes regulate the rate of cambial division in response to these seasonal shifts, whose overexpression prolongs active growth phases, while mutants show phased declines in proliferation as the tree prepares for winter dormancy [34]. Computational models of the poplar cambium further synthesize these layers, illustrating how *CLE*-*PXY* signaling dynamically maintains homeostasis. Early-phase peptide release triggers the onset of spring cambial proliferation, while mid-to-late summer feedback loops prevent over-differentiation, thus ensuring sustained and balanced wood formation throughout the annual growth cycles [36].

Together, the cambium integrates peptides originating from distinct vascular tissues to promote division while preventing over-proliferation, thereby defining narrow, continuous stem cell domains and coordinating balanced and bidirectional production of xylem and phloem [35]. The TDIF peptides, via *PXY* signaling, combine with auxin gradients and transcription factors such as *WOX4* and *HD-ZIP III* to establish a unique feedback system that ensures balance between proliferation and differentiation for sustained wood formation.

### 2.2. Cell Cycle Drivers of Cambial Proliferation

Cell cycle regulators are important drivers of cambial activity. The *CYCLIN D3* (*CYCD3*) module is key to accelerating periclinal divisions within the cambial zone, thereby directly increasing the production of vascular precursor cells [37]. Recent studies demonstrate that *CYCD3;1* acts as a critical downstream effector of cytokinin (CK) signaling to promote secondary growth, providing a conserved mechanism for regulating the rate of cambial cell proliferation [38]. In woody species, this module has expanded and diversified to meet the complex demands of perennial growth. In *Populus trichocarpa*, for instance, *PtoCYCD3*;*1* maintains its role in promoting radial growth via CK signaling, while *PtoCYCD3*;*3* has evolved additional functions in branching [21], illustrating how conserved G1 drivers are recruited for diverse woody architectural requirements.

These functional specializations are further refined by a sophisticated regulatory network, where specific *CYCD* and *CYCLIN*-*DEPENDENT KINASE* (*CDK*) family members exhibit distinct interaction preferences at the genome level to fine-tune vascular development in poplar [39]. *CDK*s are highly expressed in vascular domains including the cambium [40]. Furthermore, *CDKA;1* not only regulates cell proliferation but also couples division control to SCW biosynthesis by interacting with enzymes like UDP-glucose 6-dehydrogenase (UGDH). This interaction ensures that the supply of SCW precursors is precisely synchronized with the rate of cell production [41,42]. This integrated framework demonstrates how vascular-specific signals and hormonal cues modulate cyclin-CDK activity to coordinate tissue proliferation with the structural development of woody plants.

### 2.3. Phytohormonal Control of Cambial Activity

Phytohormones play a pivotal role in regulating cambial activity, controlling key processes such as stem cell division, fate determination, and differentiation during the secondary growth. The main phytohormones involved include auxin, CK, gibberellin (GA), ethylene (ETH), and brassinosteroid (BR), each contributing in a specific manner to the maintenance and growth of the cambium.

Auxin is a key phytohormone that transmits signals between cells in meristematic tissue, regulating various developmental behaviors [15,43]. The concentration of auxin is elevated in the cambium relative to surrounding tissues, forming a gradient that is essential for defining the organizer identity [44]. Disrupting the apical sources of auxin suppresses cambial activity and secondary xylem formation [3], underscoring the dependence of auxin gradients in the vascular pattern [45]. As the cambium grows, the position of the auxin peaks shift. *PIN*-*FORMED* (*PIN*) family transporters, which are highly expressed around the cambium, play a key role in setting auxin distribution [46]. Specifically, *PIN5* regulates intracellular levels of free indole-3-acetic acid (IAA) [46,47], controlling the availability of auxin in the cambium. Overexpression of *PdePIN5b* promotes the transport of auxin into the endoplasmic reticulum, reducing the concentration of IAA in the cytoplasm and thereby significantly suppressing cambial proliferation [47]. Auxin interacts with *TDIF*-*PXY*-*WOX* signaling to regulate the polarity of the stem cells [48]. For example, auxin upregulates *PXY* expression via *ARF5*, while *TDIF*-*PXY* signaling negatively regulates auxin response, creating a feedback loop that ensures proper cambial activity and prevents over-proliferation [15,36]. Furthermore, auxin regulates *WOX4* expression, thereby coordinating auxin fluxes and defining the stem cell organizer [14,48,49]. These dynamic gradients help maintain a narrow stem cell domain while ensuring continuous bidirectional output. Auxin signaling is also involved in regulating *HD-ZIP III*, as *PmHB1* is regulated by auxin homeostasis to affect meristem activity in *Prunus mume* [50]. Specific *ARF* members, such as *ARF6* and *ARF8*, play a more targeted role in promoting xylem formation over phloem [51], further indicating the complexity and specificity of auxin signaling components in regulating cambial activity.

CK also directly influences cambial activity by regulating cell division and radial growth. The CK biosynthesis gene *Isopentenyl transferase* (*IPT*) and the catabolic gene *Cytokinin oxidase 2* (*CKX2*) modulate cambial activity in predictable directions [52,53]. For instance, mutants of *ipt* show a loss of cambial cells, while overexpression of *PtIPT7* significantly increases the number of vascular cambial cells [52,54]. Conversely, overexpression of *CKX2* inhibits cambial activity and restricts radial growth [52], emphasizing the essential role of CK homeostasis in cambial activity. Besides that, CK receptors such as *Histidine Kinase 3* (*HK3*) and *Cytokinin Response 1* (*CRE1*) are enriched in dividing cambial cells, further supporting their role in regulating cambial proliferation [52]. Additionally, CK induces the expression of *PHLOEM EARLY DOF* (*PEAR*), which promotes procambium proliferation and patterning [55]. Downstream of cytokinin, a set of *LATERAL BOUNDARY DOMAIN* (*LBD*) transcription factors act to initiate secondary growth [56,57]. For instance, *LDB11* forms a feedback loop with reactive oxygen species (ROS) to regulate cambial proliferation, where ROS over-accumulation suppresses *LBD11* transcription, thereby ensuring balanced cambial activity [58]. This mechanism highlights the interplay between cytokinin and ROS signaling.

Auxin and cytokinin do not operate in isolation but instead interact synergistically to regulate cambial activity. A high concentration of auxin promotes xylem differentiation, while CK enhances stem cell division toward the phloem, together shaping the differentiation pattern of the cambium [59]. The interaction is underpinned by shared regulatory mechanisms, such as the *Monopteros*/*Auxin response factor 5* (*MP*/*ARF5*)-*Lonesome highway* (*LHW*)-*Target of monopteros 5* (*TMO5*) pathway, which directly drives cambial establishment and division by activating *PIN1* to establish a positive feedback loop that stabilizes polar auxin transport [12,60,61,62]. A parallel mechanism involves *LHW*-*TMO5* regulating CK, which is critical for cell proliferation and procambium patterning [63]. This interaction highlights the complementary roles of auxin and CK in regulating cambial proliferation and differentiation. Auxin signaling via *ARF*s controls cell cycle genes such as *CYCD3* and regulates lateral root initiation and cell fate changes through *AUX*/*IAA* partners [64], linking primary and secondary growth. CK further promotes cambial activity by inducing *CYCD3* expression [65], reinforcing the connection between phytohormone input and cell cycle entry. Interestingly, the role of *ARF* factors exhibits developmental context dependency. During primary vascular development, *ARF5* activates *LONELY GUY 3*/*4* (*LOG3*/*4*) to boost CK biosynthesis and promote radial division [63,66]. In contrast, during secondary growth, *ARF5* suppresses cambial activity, whereas *ARF3* and *ARF4* act positively [15]. This functional difference highlights how the same transcription factor can be reprogrammed to perform distinct roles across different developmental stages and tissue environments.

GA promotes secondary growth by increasing the accumulation of dividing cambial cells [67]. GA regulates xylem formation through the degradation of the GA signaling repressors *GIBBERELLIN INSENSITIVE* (*GAI*) and *REPRESSOR OF GA1-3* (*RGA*), promoting the differentiation of xylem vessels [51]. The promoting effect depends on *PIN1*-mediated polar auxin transport and *ARF* signaling, as its effect is inhibited in *pin1* and *arf7*/*arf19* mutants [44]. This demonstrates the interdependence of GA and auxin signaling. Additionally, the interaction between GA and auxin is facilitated by the direct activation of *WOX4* by *PtoARF7*, forming a ternary complex with DELLA and AUX/IAA to regulate cambial proliferation [68]. Besides this, the *HD*-*ZIP III* transcription factor *PHB*, expressed in the vascular tissues, reduces GA activity by increasing *GA2ox2* expression, thereby triggering endodermal accumulation of *CYCD6*;*1* that drives the formative asymmetric divisions [69].

Similarly, *ETH* enhances cambial activity through canonical signaling components and cross-talk with downstream transcriptional regulator [15,70]. Furthermore, BR increases cambial proliferation, whereas BR biosynthesis inhibition dampens secondary vascular differentiation [71], placing BR as a tunable growth amplifier in woody stems.

The phytohormonal regulation of cambial activity is dynamically modulated depending on the phase of cambial growth, integrating seasonal and environmental cues to control activation, proliferation, and senescence in woody plants. For instance, phase-dependent phytohormonal shifts are particularly evident in species like *Populus*, where phytohormones such as abscisic acid (ABA), BR, jasmonic acid (JA), and GA are upregulated during active growth phases to balance proliferation and differentiation. The expression of genes related to these phytohormones peaks mid-season, coinciding with maximal xylem production, and declines in later phases to prevent overgrowth and ensure proper tissue maturation [72]. These phytohormonal gradients interact with the *TDIF*-*PXY*-*WOX* pathway, maintaining cambial homeostasis and adapting to environmental cues such as temperature and photoperiod [73]. In *Populus*, *Pag Jasmonate ZIM-domain 5* (*PagJAZ5*), acting as a core repressor of JA signaling, coordinates CK signaling with *TYPE-A RESPONSE REGULATORS* (*ARR*) to regulate phase-specific cambial activity. During the early proliferation phase, *PagJAZ5* enhances CK sensitivity to support rapid division, while in mid-to-late phases, it promotes differentiation by repressing *PagARR*, ensuring a balanced transition between cell division and maturation and preventing premature senescence [74].

Together, these phytohormonal signals create a multilayered regulatory network that coordinates the cambial response to positional cues, proliferation signals, and growth-promoting factors. This integration is unique to cambial stem cells due to their unique role in sustaining radial growth and generating both xylem and phloem tissues in a continuous manner. Phytohormones directly regulate cambial cell division and differentiation through spatial gradients while interacting with downstream transcription factors. The crosstalk adapts cambial transitions from reactivation through peak growth to dormancy, aligning with annual cycles for both longevity and environmental resilience, thereby maintaining tissue organization and aligning the balance between self-renewal and bidirectional output required for sustained secondary growth. The temporal and spatial regulation of these pathways in response to environmental cues further sets cambial stem cells apart from other meristems in the plant.

### 2.4. Fate Determination and Differentiation

Cambial fate thresholds emerge from the interplay of spatial hormone gradients, feedback stabilized auxin transport, and gatekeeper transcription factors. Phytohormones are distributed across the radial axis, with GA enriched in developing xylem, auxin peaking in the cambium, and CK accumulating toward the phloem, thereby imparting positional information to dividing and differentiating cells [75]. Through periclinal divisions, cambial stem cells undergo radial expansion and produce daughter cells whose fates are determined by their spatial positioning relative to an organizing center [76]. This center activates *ARF5* and promotes *HD-ZIP III*, inducing proliferation in adjacent stem cells and determining the direction of cambial differentiation [77,78]. For instance, *PtrHB4* regulates the formation of the interfascicular cambium, with *Ptrhb4* mutants exhibiting a discontinuous cambium arrangement resembling a protovascular bundle pattern, which negatively affects xylem differentiation [10,79]. Similarly, *PtrHB7* is highly expressed in the cambium and modulates the balance between xylem and phloem outputs in a dosage-dependent manner. Overexpression of *PtrHB7* promotes excessive xylem formation at the expense of phloem, while the mutant displays the completely opposite phenotype [80]. In addition, *KNOTTED*-*LIKE HOMEOBOX* (*KNOX*) factors provide a complementary regulatory layer. While *AtKNOX1* maintains cambial stem cell potential in *Arabidopsis* [81], its poplar homolog *PtKNOX1* has acquired an additional function in promoting xylem fiber differentiation [20], illustrating functional innovation within woody species.

### 2.5. Cross-Meristem Commonalities

Comparative genetics across meristems reveals a conserved regulatory mechanism in which non-cell-autonomous peptide cues define the stem cell domain, and organizer transcription factors maintain stem cell identity. Both primary meristems and the cambium rely on similar signaling modules that consist of *CLE* family signaling. In the shoot apical meristem (SAM), the CLE peptide *CLAVATA3* (*CLV3*) restricts the organizer factor *WUSCHEL* (*WUS*). In turn, *WUS* promotes *CLV3* expression, establishing a dosage-sensitive feedback loop that controls the size of the stem cell domain [82,83]. A similar regulatory mechanism operates in the root apical meristem (RAM). *CLE40* signals through the receptors *CLV1* and *ACT DOMAIN CONTAINING PROTEIN 4* (*ACR4*) to position the quiescent center and limit stem cell accumulation. Meanwhile, *WOX5* functions as a root organizer analogous to *WUS* [17,84]. In contrast, the cambium exhibits more complex peptide receptor signaling to maintain stem cell identity [85]. The *CLE41* peptide promotes cambial cell division and xylem formation [86], while *CLE40* and *CLV3* inhibit stem cell division [17,82], highlighting the functional diversity of *CLE* signaling across different tissues.

*WUSCHEL* family transcription factors play a pivotal role in defining stem cell identity across these meristems. While these transcription factors share functional similarities in stem cell regulation, they operate in distinct contexts [85]. In the *SAM*, *WUS* is regulated by low auxin levels, and its role is primarily to promote stem cell proliferation and maintenance [85]. Conversely, in the cambium, *WOX4* is induced by high auxin concentrations, where it plays a critical role in maintaining cambial stem cell activity and supporting radial growth [48]. This differential auxin regulation underscores a fundamental distinction in cambial regulation, where *WOX4* is not only crucial for stem cell maintenance but also for coordinating continuous wood formation and the bidirectional production of xylem and phloem.

While both primary and secondary meristems employ conserved *CLE* peptide and *WOX* transcription factors for stem cell regulation, their functions diverge to serve different developmental objectives. In primary meristems, the *CLV3*-*CLV1*-*WUS* pathway operates through negative feedback loops, where *CLE* peptides inhibit *WUS* expression to maintain a stable stem cell reservoir [82,87]. In contrast, the *TDIF*-*PXY*-*WOX4* pathway works as a positive regulatory mechanism in the vascular cambium to promote sustained radial expansion [34,78]. The *CLE* peptides stimulate *WOX4* expression to drive stem cell division and support sustained radial growth [88]. This integration of peptide signals with auxin and other cues allows the cambium to adapt its activity over time, facilitating the continuous and bidirectional production of xylem and phloem throughout the life of the plant.

*AIL*/*PLT* transcription factors supply a further insight into the regulatory mechanisms shared across meristems. In roots, a graded distribution of *PLT*, established by *ARF*-dependent transcription, maintains high doses of stem cels and promotes transit amplifying division at lower doses, thereby repressing differentiation. The *PLT* gradient is further constrained by peptide growth factors and their receptors, reflecting similar peptide signaling axes found in the cambium [89]. In contrast, *AIL*/*PLT* functions similarly to drive proliferation and delay differentiation but operate without a global protein gradient, with *ANT* being dominant and other *PLT*s acting unequally across space and time [89]. Moreover, *ANT* is enriched in cambial stem cells, mutants reduce radial growth, and expressing *ANT* or *PLT5* from the *PXY* promoter restores cambial activity in *pxy* mutants, establishing *ANT*/*PLT* as a regulator of cambial activity in the lateral meristem [33]. These findings reveal the function of *PLT* in maintaining stem cell identity and differentiation across both primary and secondary meristems. A similar pattern is observed in the *PHYTOCHROME-INTERACTING FACTOR* (*PIF*) family. In primary growth, *PIF3*/*4*/*5* act as negative regulators of vascular patterning and xylem differentiation for auxin-mediated cell elongation but suppressing SCW formation [90,91]. In contrast, *PtoPIF3.1*/*3.2* positively regulate stem elongation and secondary xylem development through the conserved *PIF*-auxin module, while extending to modulate cambial markers like *PtoWOX4* and *PtoHB7/8* [22,92]. This functional shift highlights how gene family expansion in woody perennials adapts conserved pathways for sustained radial growth and wood formation.

Together, the conserved mechanisms across the SAM, RAM, and vascular cambium highlight both shared principles and unique adaptations in meristem regulation. *CLE* peptides, auxin signaling, and *WUSCHEL* family transcription factors are central to regulating stem cell identity and maintenance across these tissues. Additionally, the functional diversity detected in woody perennials, arising from the expansion and functional differentiation of specific gene families, has enabled the evolution of secondary growth (Table 1). These findings provide critical insights into the evolutionary adaptations that have allowed for the development of complex woody tissues and sustained perennial growth.

### 2.6. Maintenance of Cambial Stem Cells in Mature Trees

As trees age, the activity of cambial stem cells generally declines, typically resulting in reduced cell division rates and limited wood formation [100]. However, the regulatory mechanisms governing cambial aging exhibit significant interspecific variation, particularly in long-lived species where endogenous signals act to delay senescence. For instance, maintaining sufficient levels of IAA is crucial for sustaining cambial longevity [101]. In *Populus euphratica*, the upregulation of ABA, BR, and GA further assists in balancing cell proliferation with maturation [102]. Conversely, altered expressions of cell cycle regulators, such as *CYCD3*, have been shown to accelerate cambial aging in *Eucalyptus* [100]. Beyond molecular signaling, structural and functional adaptations also support sustained activity; for example, bark thickening provides thermal insulation to protect the cambium [103], while physiological shifts toward wider and longer vessels enhance hydraulic efficiency and cavitation resistance in aging stems [104].

While the ancestral bifacial cambium remains a conserved feature across most woody seed plants for the continuous production of secondary xylem and phloem, its operational longevity varies markedly between lineages. In long-lived gymnosperms such as *Ginkgo biloba*, the cambium exhibits remarkable mitotic stability that can persist for centuries. This stability is maintained through a complex interplay of reduced IAA, increased ABA, and epigenetic modulations including DNA methylation of *DAL1* and *miR166*-mediated signaling, which collectively prevent a precipitous decline in cell division [105]. Consequently, although the seasonal duration of xylem formation may shorten as these trees mature, their fundamental capacity for wood formation remains intact [24].

In contrast, angiosperms are characterized by more dynamic aging mechanisms and a broader diversity of secondary growth patterns [106]. While angiosperms like *Populus euphratica* maintain cambial homeostasis by coordinating cell expansion genes such as *XYLOGLUCAN ENDOTRANSGLUCOSYLASE*/*HYDROLASES* (*XTH*) and lignin biosynthesis pathways such as *TRANS-CINNAMATE 4-MONOOXYGENASE* (*C4H*) [102], other lineages have evolved anomalous secondary growth to adapt to specific ecological niches. For example, the development of polycyclic eusteles and successive cambia in *Commicarpus scandens* [107], along with the isolated vascular cylinders in *Manekia* [108], enable rapid biomass accumulation and architectural flexibility. These diverse strategies indicate that angiosperms utilize high degrees of transcriptional and miRNA-mediated plasticity to modulate cambial activity and longevity throughout their life cycles [102,109,110].

## 3. Xylem Development Under Cambial Regulation

### 3.1. Advances in Differentiation of Xylem

Following periclinal division, cambial daughters enter the xylem lineage through a program set by transcription factors and tuned by phytohormones [111]. For example, *Pag UNFERTILIZED EMBRYO SAC 12* (*PagUNE12*) promotes differentiation toward xylem and supports secondary vascular development [112]. Conversely, *PagMYB31*, expressed in the cambium and the early xylem, functions as a negative regulator, suppressing xylem differentiation [98]. *PttCLE47* mutants exhibit secondary xylem width and xylem cell numbers while leaving phloem largely unchanged, suggesting impaired cambial proliferation and export of cambial progeny [25]. Meanwhile, the *HD*-*ZIP III* family plays a pivotal role in cambium establishment and differentiation direction, with *Ptr Homeobox 4* (*PtrHB4*) ensuring cambial ring continuity to support normal xylem formation [10,79]. *PtrHB7* and *PtrHB8* regulate the differentiation of cambial cells into secondary xylem, and their overexpression promotes the differentiation of cambial cells towards xylem [10,113]. Furthermore, *CAIL*s regulate cambial activity and determine xylem/phloem differentiation. For example, *PLT5* rapidly inhibits the expression of xylem and phloem differentiation markers such as *CASCULAR-RELATED NAC-DOMAIN 6* (*VND6*) and *PEAR1*, while its mutants lead to the reduction in cambial cells and arresting of secondary growth [33].

Phytohormone pathways intersect with these transcriptional modules to set early differentiation thresholds. Overexpression of *IPT* simultaneously increases the contents of CK and IAA, which further promotes xylem cell output [114]. GA signaling activates IAA signaling factors such as *ARF5*/*ARF7*/*ARF19* that adjust the xylem/phloem ratio [44]. In addition, meristem identity genes enrich cell differentiation mechanisms associated with phytohormone signaling. For instance, Pto*IAA9* interacts with *PtoARF5* to inhibit the expression of *PtoHB7*/*8*, thereby inhibiting the differentiation of cambium cells into xylem [115]. Together, this coordination of transcription factors and phytohormonal signals ensures the proper differentiation and expansion of xylem cells, setting the stage for wood formation.

### 3.2. Xylem Precursor Cell Expansion

As cambial stem cells enter the xylem lineage, they progress into an elongation phase whose control is already set by earlier division and fate decisions [116,117]. Phytohormones play a central role in coordinating this transition. Auxin effector *SMALL AUXIN UP RNA* (*SAUR*) significantly promotes cell growth, highlighting a direct role for auxin in xylem expansion [118]. Similarly, GA promotes xylem precursor cell elongation, increasing vessel diameter and fiber number. This effect is consistent with phenotypes of *Gibberellin 20*-*oxidase 1* (*GA20ox1*) overexpression and DELLA degradation [119,120]. ETH further enhances xylem expansion through increasing the activity of its biosynthesis enzyme *Pt 1*-*AMINOCYCLOPROPANE*-*1*-*CARBOXYLIC ACID OXIDASE GENE 1* (*PtACO1*) and activating downstream targets such as *XTH* and *EXPANSINS* (*EXP*) through the *Pt ETHYLENE INSENSITIVE 3*-*LIKE* (*PtEIN3*) signaling pathway, leading to larger vessel and tracheid diameters [121,122]. In addition, phytohormones participate in the expansion of xylem cells by regulating the expression of cell wall-related genes and metabolic pathways to construct a multi-dimensional regulatory network. For example, *ARF5* modulates the expression of cell wall remodeling genes *EXPA1*, linking identity signaling to expansion capacity [123]. GA and auxin signals also influenced cambial cell proliferation through *WOX4*, ensuring a balanced shift between stem cell activity and differentiation [67,68].

Expansion then depends on targeted remodeling of the primary wall. Several enzymes related to the cell wall contribute to this process, with *PttEXPA1* specifically promoting radial expansion in xylem, and mutants resulting in radial growth defects [123]. *OsEXPA8* also controls endodermal anisotropy and thus influences the growth of organs [124]. Additionally, *XTH22* catalyzes xyloglucan remodeling [125]. *PECTIN METHYLESTERASES* (*PME*) further modifies wall mechanics. For example, *PME5* restricts elongation, whereas the loss of *PME35* reduces wall strength [126,127]. In parallel, *REDUCED WALL ACETYLATION 2* (*RWA2*)-mediated xylan acetylation fine-tuned wall chemistry, with *rwa2* mutants impairing fiber development and limiting xylem expansion [128].

In summary, differentiation and expansion of xylem cells arise as downstream outcomes of signals initiated in the cambium, with phytohormone inputs coupling to wall-modifying enzymes to convert early positional and identity cues into sustained cell enlargement and wood growth. This underscores the central role of cambial cell activity throughout the entire process of wood formation.

### 3.3. Secondary Cell Wall Deposition

SCW thickening plays a crucial role in translating developmental decisions into the mechanical and hydraulic properties of wood. In perennial woody plants, continuous lignin accumulation strengthens tissue and supports long-distance water transport, which together maintain growth and stability [129,130,131]. Lignin is essential for maintaining cell wall rigidity, while cellulose and hemicellulose provide tensile strength and flexibility. Specifically, cellulose microfibrils oriented in a layered pattern within the SCW impact the mechanical properties of wood [132].

Upstream cues that set cambial fate and division rates also shape SCW programs. For instance, the peptide signal *PtrCLE20* produced by xylem inhibits cambial activity in aspen, thereby indirectly affecting the number of cells that entered the wall thickening program [29]. This peptide signal restricts the initiation of SCW biosynthesis by limiting the pool of cells that can differentiate into SCW-forming xylem cells.

Several transcription factors that influence cell fate also impact SCW deposition. For example, *PagUNE12* indirectly impacts SCW thickening by influencing the timing of cambial differentiation [112]. Furthermore, loss of *PtrHB4* disrupts cambial ring continuity and compromises SCW deposition [10]. Additionally, the *Eg HISTONE VARIANT 1.3* (*EgH1.3*) and *EgMYB1* prevent premature lignification in the cambium and early xylem cells, thereby maintaining a non-lignified identity in mature parenchyma cells [133].

Three primary components of SCW are tightly regulated to ensure the mechanical and hydraulic properties of wood. For instance, *PtoMYB31* directly represses lignin biosynthesis genes, including *Caffeoyl*-*CoA*-*O*-*methyltransferase* (*CCoAOMT*), *Caffeic acid*-*O*-*methyltransferase* (*COMT*), and *Cinnamoyl alcohol dehydrogenase* (*CAD*), thereby reducing lignin deposition [98]. This regulation impacts mechanical properties by reducing the rigidity and water transport capacity of wood. Furthermore, *ARF3* and *ARF5* modulate the expression of *Cellulose synthase* (*CesA*), which is involved in cellulose biosynthesis during cambial differentiation [134]. Additionally, *WOX4* modulates the expression of hemicellulose-related genes, coordinating the deposition of hemicellulose in the secondary wall during cambial differentiation [135].

Therefore, SCW deposition functions as a late-stage developmental program that precedes programmed cell death. It remains tightly coupled to earlier cambial regulation, where upstream signals modulate cambial activity and influence cell fate decisions, propagating forward to determine wall thickness, composition, and ultimately wood formation. These regulatory processes ensure that cellulose, hemicellulose, and lignin are precisely coordinated to optimize the physical properties of wood. The regulatory genes involved in wood formation in woody plants are summarized in Table 2, which highlights key genes responsible for regulating cambial activity, cell differentiation, and secondary wall biosynthesis.

## 4. Frontiers and Future Directions

### 4.1. Single-Cell, Spatial Transcriptomics, and Quantitative Modeling

**Figure 2:**
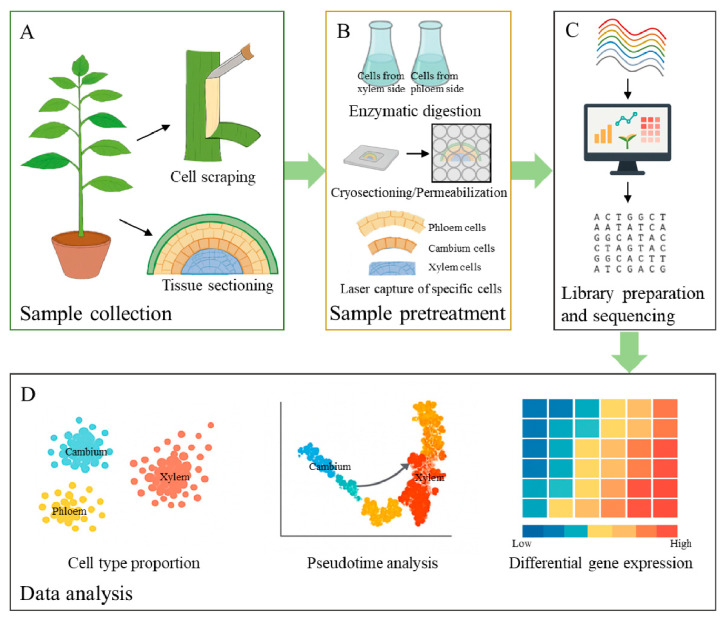
Workflow from sample collection to data-driven inference of cambial regulation. (**A**) Sample collection. Stem tissues are harvested. Bark is peeled to expose the vascular cambium for tissue scraping, and transverse sections reveal phloem, cambium, and xylem. (**B**) Sample pretreatment. Three complementary approaches are illustrated: firstly, enzymatic digestion of tissues from the xylem and phloem sides is carried out separately to obtain protoplasts for single-cell sequencing; cryosection is performed followed by permeabilization for spatial transcriptomics. In addition, specific cell types are captured using laser for detailed tissue detection. (**C**) Library preparation and sequencing. Isolated cells or tissues are processed for RNA-seq, generating expression profiles for downstream analyses. (**D**) Data analysis. Representative outputs include: cell-type composition and clustering (left), pseudotime trajectories from cambium to phloem or xylem (middle), and differential gene expression heat maps (right) highlighting gradients and lineage-specific programs.

Traditional bulk transcriptomics and histology have faced challenges, including low resolution in capturing cellular heterogeneity, spatial gradients, and dynamic interactions, often masking rare subpopulations or transient states critical for fate decisions [137]. Emerging single-cell and spatial transcriptomic approaches provide high resolution insights into cambial heterogeneity and differentiation trajectories in woody species (Figure 2). These advanced approaches provide new empirical data on spatial gradients and cell subpopulations, which bridge molecular inputs to cambial outputs.

Single-cell RNA sequencing in the SAM vascular differentiation captures low-abundance transcripts in the cell nucleus, revealing differentiation pathways in the xylem and phloem and linking *TDIF*-*PXY*-*WOX4* signaling to asymmetric fate determination during cambial division, while identifying specific cell subpopulations essential for vascular tissue specialization and wood formation [138]. Similarly, a transcriptional landscape of stems identifies distinct cell clusters and further reveals cellular heterogeneity and lineage progression at single-cell resolution [139]. These approaches have revealed the occurrence and maintenance of cell heterogeneity within the cambium. Complementing cellular resolution, high-spatial-resolution transcriptome profiles have consecutive radial sections across the phloem, cambium, and xylem, providing a positional reference for wood-forming domains, thus identifying key markers and their expression gradients, and further clarifying the regional differences in the activity and differentiation of the cambium [140]. Spatial transcriptome analyses identify two distinct types of meristematic-like cell pools within secondary vascular tissues, thereby directly linking positional information to developmental progression and refining models of cambial organization and differentiation [141]. Furthermore, combining single-cell RNA sequencing and spatial transcriptome analyses localizes cambial sub-domains and expression gradients of key regulators, revealing distinct expression patterns of key factors like *WOX4* and *HD*-*ZIP III* across different cambial subpopulations [142,143]. This integration has revealed distinct patterns of gene expression that are crucial for regulating cambial proliferation and differentiation, linking cellular behaviors to spatial positioning within the cambial zone. It also demonstrates how signal gradients and transcriptional programs are spatially organized to guide tissue patterning, offering potential solutions for cambial dynamics. Additionally, a stem-differentiating xylem (SDX) protoplast workflow, coupling RNA-seq with ChIP-seq, provides a strategy to detect hierarchical regulatory networks, linking upstream signals like *ARF5* to downstream xylem development [144].

Laser capture microdissection (LCM) combined with transcriptomes has been pivotal for further interpreting the information that constitutes the map in specific tissues. LCM-guided analyses show that high CK levels in the phloem promote cambial proliferation, confirming its key role in cambial development [52]. Meanwhile, an auxin maximum centers on the cambium, providing quantitative inputs for fate and division models [59]. Focusing LCM on defined cambial cell layers enables discovery of tissue-restricted gene programs. Notably, 95 cambium-specific transcription factors are identified, which illustrates the precision of LCM in isolating gene expression patterns [78]. The tissue-specific analysis provides a tool for dissecting the complex molecular mechanisms that underlie wood formation, bridging the gap between cellular resolution and tissue-level functional analysis.

Moreover, quantitative modeling provides a mechanistic bridge from measured inputs to predicted cambial behaviors, converting inter-molecular interactions into direct developmental outcomes. Mathematical models integrate the complex interactions between *PXY* and *MP* signaling in cambial development, revealing how diverging interactions between these pathways impact stem cell activity and tissue patterning during growth [145]. Additionally, with the detection of more high-resolution data, frameworks that parameterize peptide signals, phytohormone signals, and transcription factor thresholds can predict the width of the cambial stem cell domain and the boundary of division and differentiation, as well as simulate cambial dynamics under various physiological conditions through running perturbations [33].

Integrating single-cell, spatial, and LCM-derived transcriptome with quantitative models improves prediction of cambial dynamics and guides experimental design and trait targeting. These advanced techniques not only map gene expression but also enable reconstruction of regulatory networks and dynamic gradient formation [78,142]. Coupled with mathematical models that simulate feedback loops and diffusion processes, they provide a mechanistic understanding of how gene families expand and diversify to generate phenotypic variation in wood development [98], informing strategies to optimize wood formation.

### 4.2. Epigenetic Regulation

Chromatin state and protein modifications work together to fine-tune wood formation. In *Eucalyptus*, a linker histone and TF complex, *EgH1.3*-*EgMYB1*, directly represses lignin biosynthetic genes, prevents premature lignification in the cambium and early xylem, and maintains a non-lignified identity in mature parenchyma [133]. Cambium identity is also set by chromatin state. *PtrVCS2* modulates histone acetylation at *PtrWOX4a*, thereby adjusting the cambial layer number and secondary growth [78]. Beyond chromatin, post-translational control limits cambial division. DA1-type ubiquitin-associated proteins reduce *WOX4* stability and restrain cambial proliferation, establishing a protein-level brake on stem cell activity [146]. Methodologically, applying chromatin profiling assays such as an assay for transposase-accessible chromatin with high throughput sequencing (ATAC-seq) and cleavage under targets and tagmentation (CUT&Tag) to cambium-enriched tissues should connect chromatin accessibility and histone marks to stem cell potential, identity gating, and lineage bias, thereby complementing transcriptional atlases [144].

### 4.3. Non-Coding RNAs

Non-coding RNAs add regulatory layers that interface with hormone pathways and transcription factor networks to shape cambial programs. A general splicing factor, *CELL DIVISION CYCLE 5* (*CDC5*), promotes pri-miRNA transcription and processing, with mutants reducing multiple miRNAs including *miR165*, *miR166*, and *miR167* [147]. This places miRNA dosage upstream of core developmental transcription factors and suggests system-wide consequences for cambial regulation. Within this framework, *miR165*/*166* dosage modulates the width and position of the cambial identity zone by adjusting *HD*-*ZIP III* activity [143]. A second module links miRNA to phytohormone through *miR167* targeting *ARF*s. In *cdc5-1* mutants, derepressing of *ARF3*/*8* illustrates the sensitivity of the auxin-response machinery to miRNA dosage [147]. As *ARF5* promoting CK biosynthesis and CK-induced *CYCD3* drives cambial proliferation, *miRNA167*-mediated control of *ARF* dosage provides a route to couple the miRNA layer with multiple phytohormone signaling pathways and transcription factor regulatory networks [15,37,65].

Long non-coding RNAs (lncRNAs) appear to operate with miRNAs in a complementary manner. Mechanistically, lncRNAs act in cis and in trans, linking positional information to stable changes in gene activity that are distinct from miRNA-mediated mRNA cleavage or translational control [148]. A multi-tissue transcriptome catalogs 2988 high-confidence lncRNAs with tissue-oriented expression, pointing to organ- and tissue-specific developmental programs relevant to cambial identity and differentiation patterning [149].

Together, non-coding RNAs constitute a regulatory layer that interfaces with peptide, phytohormone, and transcription factor networks. Their dosage and spatial distribution likely influence stem cell domain size and differentiation output, with direct consequences for wood quantity and quality.

## 5. Conclusions and Perspective

Cambial stem cell activity is a dynamic process integrated through peptide signaling [31], transcription factor networks [59], and phytohormone gradients [46]. In woody plants, these elements operate within a cylindrical lateral meristem that must preserve circumferential continuity while delivering balanced xylem and phloem output [10,150]. The cambium is not a static structure but a responsive system where upstream signals continuously define the localization and maintenance of stem cells, while downstream programs determine the expansion of precursors and the deposition of SCW during cell differentiation [33,151]. This dynamic balance is essential for maintaining both the growth and functional adaptability of trees in response to fluctuating environmental conditions. Additionally, epigenetic regulators, protein turnover, and non-coding RNAs provide additional layers of complexity, refining the decisions that govern cambial behavior across diverse developmental stages [146,147].

However, a significant challenge remains in bridging the gap between molecular insights obtained from herbaceous models like *Arabidopsis* and their application to woody plants. The morphology and activity of xylem in woody plants are different from those in *Arabidopsis*. Notably, woody plants exhibit lineage-specific expansion and functionalization of key gene families (Table 1), which enriches the complexity of secondary development of woody plants. Furthermore, as perennial plants, trees face unique temporal constraints. For example, cambial activity varies dramatically with the seasons, with the number of cambial cell layers reaching dozens in summer but retreating to only a few in winter [100,105,152]. Moreover, the age difference in trees significantly influences cambial activity [105]. Deciphering how the cambium maintains its stem cell reservoir and adjusts its output in response to these seasonal and age-related variations remains a critical frontier for further investigation.

With the advancement of single-cell and spatial omics, integrative epigenomics, and quantitative modeling, we can enable a transition from merely describing cambial dynamics to predicting and designing cambial behavior in woody plants [153]. These emerging technologies hold great potential for guiding rational breeding strategies to enhance wood biomass accumulation.

In summary, the multilayered regulation of cambial stem cells, driven by dynamic feedback mechanisms, is the key to optimizing wood formation. Future studies should focus on fine-tuning these regulatory networks, including adjusting peptide signaling, phytohormone levels, and transcriptional factors, to balance proliferation and differentiation for improved wood properties [33,154]. The layered mechanism in Figure 1 and the analytical workflow in Figure 2 form a synergistic loop: the cell type profiles, spatial gradients, and regulatory nodes identified via high-resolution omics (Figure 2) calibrate the peptide hormone effector thresholds (Figure 1), ultimately providing a roadmap for optimizing traits for sustainable material production and bioenergy applications.

## Figures and Tables

**Table 1:** Lineage-specific expansion and functional innovation of gene families in woody plants compared with *Arabidopsis*.

Gene Family	Arabidopsis Representative and Function	Woody Plant Representative and Expanded Function
*CLE*	*CLE41*/*44* from phloem produce TDIF, promoting cambial division and ring continuity [31].	*PttCLE47* expressed in cambium promotes proliferation; xylem-derived *PtrCLE20* suppresses division [25,29].
*CYCD*	*CYCD3* promotes cambial division [37].	*PtCYCD3;1* responds to CK and promotes radial growth; *PtCYCD3;3* participates in branching [21].
*GRF*	*GRF*s regulate organ growth and cell proliferation.	*PtGRF*s additionally transform into regulating secondary development.
*HD-ZIP III*	*PHB*/*PHV*/*REV* pattern vascular polarity and xylem identity [93].	*PtrHB4* ensures ring continuity [10]; *PtrHB7*/*8* balances xylem/phloem production at different dosages [80].
*KNOX*	*KNAT1* maintains cambium activity [94].	*PtKNOX1* additionally promotes fiber differentiation [20].
*MYB*	*MYB46*/*83* are the main regulators of SCW biosynthesis [95].	*PtrMYB3*/*20* are functionally homologous and have the same function [96,97]; *PagMYB31* simultaneously regulates the development of cambium and xylem [98].
*PIF*	*PIF3/4/5* inhibits vascular development and SCW formation under shade [90].	*PtoPIF3.1*/*3.2* promotes stem elongation, vascular division, and secondary xylem development by activating the auxin pathway [22]; *PIF4a*/*PIF4.1* causes leaf curling and inhibits growth; *PIF8* responds to seasonal signals and mediates the cessation of growth under short-day conditions [99].
*WOX*	*WOX4*/*13*/*14* maintain cambial activity [13]; *WOX5* maintains RAM stem cell niche [84].	Lacking the direct homologous gene of *WOX14*; *PtrWOX13* promotes lignification [78]; *PtWOX5* increases adventitious roots and participates in root architecture [19].

**Table 2:** Key regulatory genes involved in cambial activity and wood formation in woody plants.

Category	Gene Name	Function	Studied Plant Species	Reference
Peptide signaling	*CLE41/42/44*	Processes into TDIF peptides, which regulates cambial activity via non-cell-autonomous communication	*Populus trichocarpa* *Populus tremula* × *tremuloides* *Betula pendula*	[25,29,35]
	*CLE47*	Impairs cambial proliferation	*Populus tremula* × *tremuloides*	[25]
	*WOX4*	Cambial division	*Populus trichocarpa* *Betula pendula*	[34,35,78]
*Cell proliferation and cycle control*	*CYCD3*	Cambial division	*Prunus mume* *Populus trichocarpa*	[21,136]
	*LBD11*	Regulates cambial proliferation through feedback with ROS signaling	*Populus tremula* × *alba*	[57]
*Hormonal signaling*	*ACO1*	Regulates ETH biosynthesis; enhances xylem expansion	*Populus tremula* × *tremuloides*	[121]
	*ARF5*/7	Adjusts xylem/phloem ratio; activates *WOX4*	*Populus tomentosa*	[115]
	*EIN3*	Activated by ETH; regulates downstream targets like *XTH* and *EXP* for xylem expansion	*Populus tremula* × *tremuloides*	[122]
	*HB1*	Regulates auxin homeostasis to affect meristem activity	*Prunus mume*	[50]
	*IPT7*	Increases the number of vascular cambial cells	*Betula pendula* *Populus trichocarpa* *Populus tremula* × *tremuloides*	[52,114]
	*JAZ5*	Coordinates CK signaling to regulate cambial activity	*Populus alba* × *Populus glandulosa*	[74]
	*PIF8*	Modulates the transition between cambial dormancy and activation in response to changing day length	*Populus tremula × tremuloides*	[99]
	*PIN5*	Regulates intracellular levels of free IAA	*Populus deltoides* × *euramericana*	[47]
*Xylem differentiation and cell wall modification*	*C4H*	Lignin biosynthesis	*Populus euphratica*	[102]
	*EXP*	Cell expansion	*Populus euphratica* *Populus tremula* × *tremuloides*	[102]
	*HB4/7/8*	Regulates differentiation of cambial cells into secondary xylem	*Populus* × *euramericana* cv. ‘Nanlin895’	[10]
	*H1.3*	Prevents premature lignification in cambium and early xylem cells	*Eucalyptus*	[133]
	*MYB1*	Inhibits premature lignification, regulating lignin deposition	*Eucalyptus*	[133]
	*MYB31*	Suppresses xylem formation	*Populus alba* × *glandulosa*	[98]
	*UNE12*	Influences SCW thickening by affecting the timing of cambial differentiation	*Populus alba* × *glandulosa*	[112]
	*XTH*	Cell expansion	*Populus euphratica*	[102]

## Data Availability

Data sharing is not applicable to this article as no datasets were generated or analyzed during the current study.

## Abbreviations

The following abbreviations are used in this manuscript:

ABA	Abscisic acid
ACO1	1-AMINOCYCLOPROPANE-1-CARBOXYLIC ACID OXIDASE GENE 1
ACR4	ACT DOMAIN CONTAINING PROTEIN 4
ARF	Auxin Response Factor
ARR	TYPE-A RESPONSE REGULATORS
ATAC-seq	Assay for Transposase-Accessible Chromatin with high-throughput sequencing
BR	Brassinosteroid
C4H	TRANS-CINNAMATE 4-MONOOXYGENASE
CAD	Cinnamoyl alcohol dehydrogenase
CAIL	CAMBIUM-EXPRESSED AINTEGUMENTA-LIKE
CCoAOMT	Caffeoyl-CoA-O-methyltransferase
CDC5	CELL DIVISION CYCLE 5
CDK	CYCLIN-DEPENDENT KINASE
CesA	Cellulose synthase
CK	Cytokinin
CKX2	Cytokinin oxidase 2
CLE	CLAVATA3/EMBRYO SURROUNDING REGION
COMT	Caffeic acid-O-methyltransferase
CRE1	Cytokinin Response 1
CUT&Tag	Cleavage Under Targets and Tagmentation
CYCD	D-type Cyclin
EIN3	ETHYLENE INSENSITIVE 3
ETH	Ethylene
EXP	EXPANSINS
GA	Gibberellin
GAI	GIBBERELLIN INSENSITIVE
GRF	GROWTH-REGULATING FACTOR
HD-ZIP III	Class III Homeodomain-Leucine Zipper
HK3	Histidine Kinase 3
IAA	Indole-3-acetic acid
IPT	Isopentenyl transferase
JA	Jasmonic acid
JAZ5	Jasmonate ZIM-domain 5
KNAT1	KNOTTED-like from Arabidopsis thaliana 1
KNOX	KNOTTED-LIKE HOMEOBOX
LBD	LATERAL BOUNDARY DOMAIN
LCM	Laser Capture Microdissection
LHW	Lonesome highway
lncRNA	Long non-coding RNA
LOG	LONELY GUY
PEAR	PHLOEM EARLY DOF
PIF	PHYTOCHROME-INTERACTING FACTOR
PIN	PIN-FORMED
PME	PECTIN METHYLESTERASES
PXY	PHLOEM INTERCALATED WITH XYLEM
RAM	Root Apical Meristem
RGA	REPRESSOR OF GA1-3
ROS	Reactive Oxygen Species
RWA2	REDUCED WALL ACETYLATION 2
SAM	Shoot Apical Meristem
SAUR	SMALL AUXIN UP RNA
SCW	Secondary Cell Wall
SDX	Stem-differentiating xylem
TDIF	TRACHEARY ELEMENT DIFFERENTIATION INHIBITORY FACTOR
TMO5	Target of monopteros 5
UGDH	UDP-glucose 6-dehydrogenase
VND6	VASCULAR-RELATED NAC-DOMAIN 6
WOX	WUSCHEL-RELATED HOMEOBOX
WUS	WUSCHEL
XTH	XYLOGLUCAN ENDOTRANSGLUCOSYLASE/HYDROLASES
